# Protein Type, Protein Dose, and Age Modulate Dietary Protein Digestion and Phenylalanine Absorption Kinetics and Plasma Phenylalanine Availability in Humans

**DOI:** 10.1093/jn/nxaa024

**Published:** 2020-02-18

**Authors:** Stefan H M Gorissen, Jorn Trommelen, Imre W K Kouw, Andrew M Holwerda, Bart Pennings, Bart B L Groen, Benjamin T Wall, Tyler A Churchward-Venne, Astrid M H Horstman, René Koopman, Nicholas A Burd, Cas J Fuchs, Marlou L Dirks, Peter T Res, Joan M G Senden, Jan M J M Steijns, Lisette C P G M de Groot, Lex B Verdijk, Luc J C van Loon

**Affiliations:** 1 Department of Human Biology, NUTRIM School of Nutrition and Translational Research in Metabolism, Maastricht University Medical Centre+ (MUMC+), Maastricht, Netherlands; 2 FrieslandCampina, Amersfoort, Netherlands; 3 Division of Human Nutrition, Wageningen University, Wageningen, Netherlands

**Keywords:** healthy aging, muscle protein synthesis, muscle mass maintenance, splanchnic extraction, sarcopenia

## Abstract

**Background:**

Dietary protein ingestion stimulates muscle protein synthesis by providing amino acids to the muscle. The magnitude and duration of the postprandial increase in muscle protein synthesis rates are largely determined by dietary protein digestion and amino acid absorption kinetics.

**Objective:**

We assessed the impact of protein type, protein dose, and age on dietary protein digestion and amino acid absorption kinetics in vivo in humans.

**Methods:**

We included data from 18 randomized controlled trials with a total of 602 participants [age: 53 ± 23 y; BMI (kg/m^2^): 24.8 ± 3.3] who consumed various quantities of intrinsically l-[1-^13^C]-phenylalanine–labeled whey (*n* = 137), casein (*n* = 393), or milk (*n* = 72) protein and received intravenous infusions of l-[ring-^2^H_5_]-phenylalanine, which allowed us to assess protein digestion and phenylalanine absorption kinetics and the postprandial release of dietary protein–derived phenylalanine into the circulation. The effect of aging on these processes was assessed in a subset of 82 young (aged 22 ± 3 y) and 83 older (aged 71 ± 5 y) individuals.

**Results:**

A total of 50% ± 14% of dietary protein–derived phenylalanine appeared in the circulation over a 5-h postprandial period. Casein ingestion resulted in a smaller (45% ± 11%), whey protein ingestion in an intermediate (57% ± 10%), and milk protein ingestion in a greater (65% ± 13%) fraction of dietary protein–derived phenylalanine appearing in the circulation (*P* < 0.001). The postprandial availability of dietary protein–derived phenylalanine in the circulation increased with the ingestion of greater protein doses (*P* < 0.05). Protein digestion and phenylalanine absorption kinetics were attenuated in older when compared with young individuals, with 45% ± 10% vs. 51% ± 14% of dietary protein–derived phenylalanine appearing in the circulation, respectively (*P* = 0.001).

**Conclusions:**

Protein type, protein dose, and age modulate dietary protein digestion and amino acid absorption kinetics and subsequent postprandial plasma amino acid availability in vivo in humans. These trials were registered at clinicaltrials.gov as NCT00557388, NCT00936039, NCT00991523, NCT01317511, NCT01473576, NCT01576848, NCT01578590, NCT01615276, NCT01680146, NCT01820975, NCT01986842, and NCT02596542, and at http://www.trialregister.nl as NTR3638, NTR3885, NTR4060, NTR4429, and NTR4492.

See corresponding commentary on page 2001.

## Introduction

Dietary protein ingestion stimulates muscle protein synthesis rates by providing amino acids to the muscle (1, 2). Various factors have been shown to influence the postprandial muscle protein synthetic response, such as dietary protein digestion and amino acid absorption (3, 4), splanchnic amino acid retention (5, 6), plasma amino acid availability, muscle perfusion and the delivery of amino acids to the muscle (7, 8), uptake of amino acids by the muscle (9, 10), and intramuscular signaling that results in the activation of the muscle protein synthetic machinery (11, 12). Consequently, the ingested protein amount (13–15), source (16–18), and type (3, 19, 20) differentially impact plasma amino acid profiles and, as such, the magnitude and duration of the postprandial increase in muscle protein synthesis rates.

Ingestion of whey protein has been shown to strongly increase muscle protein synthesis rates over a short(er) time frame, whereas casein ingestion may stimulate muscle protein synthesis rates to a moderate extent for a more prolonged time period (3). The divergent postprandial muscle protein synthetic responses seem to be, at least partly, attributed to the more rapid protein digestion after ingestion of whey compared with micellar casein (3, 4). Although whey protein and casein have been studied extensively (3, 4, 14–17, 19–22), less is known regarding the dietary protein digestion and amino acid absorption kinetics of intact bovine milk protein, providing both whey and casein in a ratio of ∼20:80. Besides protein type, ingesting a greater amount of protein generally results in a dose-dependent stimulation of muscle protein synthesis until a plateau is reached (14, 15, 23, 24). Whether the protein dose affects the postprandial release of dietary protein–derived amino acids into the circulation and whether the dose–response in postprandial amino acid availability is specific for each type of protein have not been clearly established.

Another factor determining the muscle protein synthetic response to protein ingestion is age. Older age is associated with a lower muscle protein synthetic response to food ingestion, also referred to as anabolic resistance (11, 25). Anabolic resistance to protein intake is believed to be one of the factors responsible for the loss of muscle mass observed with increasing age (11, 25, 26). It has been suggested that impairments in dietary protein digestion and amino acid absorption kinetics, resulting in increased splanchnic amino acid retention and a blunted postprandial increase in circulating amino acids, may contribute to age-related anabolic resistance of muscle protein synthesis rates. While some studies suggest greater splanchnic amino acid retention following amino acid administration in older compared with younger adults (5, 6), other studies have failed to confirm those findings following the ingestion of a single bolus of protein (27, 28). Thus, it remains unclear whether protein digestion and amino acid absorption kinetics are impaired with aging.

Over the past decade, we have conducted 18 randomized controlled trials (RCTs) in which we applied intravenous infusions of l-[ring-^2^H_5_]-phenylalanine in combination with the ingestion of intrinsically l-[1-^13^C]-phenylalanine–labeled protein to assess dietary protein digestion and phenylalanine absorption kinetics, as well as the subsequent plasma availability of dietary protein–derived phenylalanine in young and older individuals ingesting varying amounts of casein, whey, or milk protein concentrate (20, 21, 24, 27, 29–48). Combining data from 18 RCTs, with >600 participants who underwent the same experimental procedures, provided us with an ideal opportunity to assess the impact of protein type (i.e., casein, whey, and milk protein), protein dose, and age on protein digestion and phenylalanine absorption kinetics, and the subsequent postprandial release of dietary protein–derived phenylalanine into the circulation in vivo in humans.

## Methods

### Data selection

The present study includes data from 18 previously published RCTs from our laboratory (20, 21, 24, 27, 29–49). All RCTs were approved by the Medical Ethics Committee of Maastricht UMC+ and the procedures followed were in accordance with the latest version of the Helsinki Declaration. The included studies involved oral consumption of a single dose of intrinsically l-[1-^13^C]-phenylalanine–labeled protein, in combination with a primed, constant l-[ring-^2^H_5_]-phenylalanine infusion to determine dietary protein digestion and amino acid absorption kinetics and plasma amino acid availability over ≥3 h. Studies, or study arms, that used hydrolyzed protein or beef protein were not included in the analysis, which resulted in the inclusion of 602 participants who consumed various quantities of intact whey, micellar casein, or milk protein. The majority of participants were men, with only a few women (*n* = 12) included from a recent study in which we showed that dietary protein digestion and amino acid absorption kinetics do not differ between men and women (49).

To assess the effects of protein type on dietary protein digestion and amino acid absorption kinetics and postprandial plasma amino acid availability, we included data from all 602 participants who consumed either whey protein (*n* = 137), casein (*n* = 393), or milk protein concentrate (*n* = 72; see **Table 1** for participants’ characteristics). To investigate the effects of protein dose on dietary protein digestion and amino acid absorption kinetics and postprandial plasma amino acid availability, we conducted separate analyses for whey protein and casein. Only studies that measured protein digestion and amino acid absorption kinetics for ≥5 h after protein ingestion were included. This approach resulted in *n* = 76 for whey protein and *n* = 359 for casein. Protein dose was expressed in grams of protein per kilogram of body mass (BM), and participants were divided in tertiles (T) of protein dose (see **Tables 2** and **3** for participants’ characteristics). The use of relative protein intakes is preferred over absolute protein intakes as this takes into account the differences in BM between individuals (23, 50). Whey protein dose ranged from 0.15 to 0.27 g/kg BM for T1, 0.28 to 0.30 g/kg BM for T2, and 0.31 to 0.46 g/kg BM for T3. Casein dose ranged from 0.20 to 0.25 g/kg BM for T1, 0.26 to 0.33 g/kg BM for T2, and 0.34 to 0.64 g/kg BM for T3. Finally, for the comparison between young and older individuals, we included 4 RCTs that compared young and older individuals all within the RCT. These 4 studies were conducted in healthy young and older men ingesting 35 g (27) or 20 g (32, 35, 42) intrinsically labeled casein, measured for ≥5 h, and included experimental arms that also involved exercise (42), carbohydrate co-ingestion (32), or local insulin infusion (35). This approach yielded data from *n* = 82 young and *n* = 83 older individuals (see **Table 4** for participants’ characteristics).

**TABLE 1:** Characteristics of participants who consumed intrinsically labeled whey, casein, or milk protein^1^

	Whey (*n* = 137)	Casein (*n* = 393)	Milk (*n* = 72)
Dose			
g	23 ± 5^a^	24 ± 7^a,b^	26 ± 11^b^
g/kg BM	0.29 ± 0.08^a^	0.32 ± 0.10^a,b^	0.33 ± 0.13^b^
g/kg LBM	0.38 ± 0.11^a^	0.40 ± 0.12^a,b^	0.43 ± 0.18^b^
Age, y	54 ± 20	52 ± 24	51 ± 22
Men/women, *n*/*n*	125/12	393/0	72/0
Height, m	1.77 ± 0.07	1.78 ± 0.07	1.79 ± 0.07
BM, kg	80.3 ± 15.7^a^	77.5 ± 9.1^b^	78.5 ± 9.3^a,b^
BMI, kg/m^2^	25.6 ± 4.5^a^	24.6 ± 2.8^b^	24.5 ± 3.4^a,b^
Fat, %	21.6 ± 5.9^a^	19.1 ± 4.9^b^	20.1 ± 5.4^a,b^
ALM, kg	27.9 ± 5.1	27.2 ± 3.5	26.8 ± 3.7
LBM, kg	61.0 ± 8.8	60.7 ± 6.2	60.3 ± 6.7
HbA1c, %	5.5 ± 0.5^a,b^	5.7 ± 0.7^a^	5.4 ± 0.3^b^
Plasma glucose, mmol/L			
Fasting	5.5 ± 0.6^a^	5.9 ± 1.4^b^	5.9 ± 0.6^b^
2 h post–75-g oral-glucose	5.8 ± 1.8^a^	6.7 ± 3.5^b^	5.8 ± 1.6^a,b^

1 Values are means ± SDs. Labeled means (in a row) without a common letter differ, *P* < 0.05. ALM, appendicular lean mass; BM, body mass; HbA1c, glycated hemoglobin; LBM, lean body mass.

**TABLE 2:** Characteristics of participants who consumed a low, medium, or high dose of intrinsically labeled whey protein^1^

	T1 (*n* = 25)	T2 (*n* = 25)	T3 (*n* = 26)
Dose			
g	22 ± 2^a^	23 ± 2^b^	24 ± 1^b^
g/kg BM	0.23 ± 0.04^a^	0.29 ± 0.01^b^	0.35 ± 0.04^c^
g/kg LBM	0.32 ± 0.04^a^	0.38 ± 0.03^b^	0.47 ± 0.05^c^
Age, y	59 ± 13	59 ± 13	52 ± 11
Men/women, *n*/*n*	25/0	25/0	14/12
Height, m	1.78 ± 0.08^a^	1.79 ± 0.08^a^	1.72 ± 0.05^b^
BM, kg	99.3 ± 24.3^a^	79.7 ± 6.6^b^	68.6 ± 8.6^c^
BMI, kg/m^2^	31.4 ± 6.6^a^	25.0 ± 1.9^b^	23.0 ± 2.3^b^
Fat, %	27.4 ± 7.0^a^	20.9 ± 4.6^b^	23.3 ± 4.0^b^
ALM, kg	33.5 ± 4.8^a^	28.7 ± 2.5^b^	22.8 ± 3.4^c^
LBM, kg	69.4 ± 10.3^a^	61.1 ± 4.4^b^	50.7 ± 6.8^c^
HbA1c, %	5.8 ± 0.4^a^	5.5 ± 0.5^a,b^	5.3 ± 0.3^b^
Plasma glucose, mmol/L			
Fasting	6.0 ± 0.7^a^	5.5 ± 0.5^b^	5.4 ± 0.5^b^
2 h post–75-g oral-glucose	6.6 ± 2.0^a^	5.2 ± 1.5^b^	5.0 ± 1.7^b^

1 Values are means ± SDs. Labeled means (in a row) without a common letter differ, *P* < 0.05. Protein dose ranged from 0.15 to 0.27 g/kg BM for T1, 0.28 to 0.30 g/kg BM for T2, and 0.31 to 0.46 g/kg BM for T3. ALM, appendicular lean mass; BM, body mass; HbA1c, glycated hemoglobin; LBM, lean body mass; T, tertile.

**TABLE 3:** Characteristics of participants who consumed a low, medium, or high dose of intrinsically labeled casein^1^

	T1 (*n* = 120)	T2 (*n* = 120)	T3 (*n* = 119)
Dose			
g	20 ± 1^a^	21 ± 2^a^	33 ± 7^b^
g/kg BM	0.24 ± 0.01^a^	0.28 ± 0.02^b^	0.44 ± 0.07^c^
g/kg LBM	0.31 ± 0.02^a^	0.36 ± 0.04^b^	0.55 ± 0.11^c^
Age, y	60 ± 21^a^	56 ± 24^a^	46 ± 25^b^
Men/women, *n*/*n*	120/0	120/0	119/0
Height, m	1.79 ± 0.07^a^	1.76 ± 0.07^b^	1.78 ± 0.07^a,b^
BM, kg	84.1 ± 5.4^a^	73.3 ± 6.7^b^	73.4 ± 9.4^b^
BMI, kg/m^2^	26.5 ± 2.1^a^	23.7 ± 2.3^b^	23.3 ± 2.8^b^
Fat, %	21.1 ± 4.0^a^	18.5 ± 4.9^b^	17.0 ± 5.0^b^
ALM, kg	27.6 ± 2.8^a^	25.2 ± 2.5^b^	27.0 ± 3.9^a^
LBM, kg	64.5 ± 4.8^a^	57.9 ± 4.2^b^	58.4 ± 7.0^b^
HbA1c, %	5.9 ± 0.9^a^	5.6 ± 0.4^b^	5.6 ± 0.4^b^
Plasma glucose, mmol/L			
Fasting	6.3 ± 1.9^a^	5.7 ± 0.8^b^	5.7 ± 0.7^b^
2 h post–75-g oral-glucose	7.8 ± 4.7^a^	6.1 ± 2.4^b^	6.0 ± 1.5^b^

1 Values are means ± SDs. Labeled means (in a row) without a common letter differ, *P* < 0.05. Protein dose ranged from 0.20 to 0.25 g/kg BM for T1, 0.26 to 0.33 g/kg BM for T2, and 0.34 to 0.64 g/kg BM for T3. ALM, appendicular lean mass; BM, body mass; HbA1c, glycated hemoglobin; LBM, lean body mass; T, tertile.

**TABLE 4:** Characteristics of young (age 22 ± 3 y) and older (age 71 ± 5 y) men who consumed intrinsically labeled casein^1^

	Young (*n* = 82)	Older (*n* = 83)
Dose		
g	22 ± 5	22 ± 5
g/kg BM	0.30 ± 0.07	0.29 ± 0.07
g/kg LBM	0.33 ± 0.04	0.34 ± 0.04
Age, y	22 ± 3	71 ± 5*
Height, m	1.82 ± 0.07	1.75 ± 0.06*
BM, kg	74.2 ± 9.1	77.1 ± 9.4*
BMI, kg/m^2^	22.4 ± 2.2	25.2 ± 2.6*
Fat, %	15.2 ± 3.5	20.0 ± 3.7*
ALM, kg	27.0 ± 3.3	25.6 ± 3.0*
LBM, kg	60.9 ± 7.3	59.7 ± 6.1
HbA1c, %	5.3 ± 0.3	5.7 ± 0.4*
Plasma glucose, mmol/L		
Fasting	5.1 ± 0.3	5.6 ± 0.5*
2 h post–75-g oral-glucose	4.5 ± 1.0	6.1 ± 1.6*

1 Values are means ± SDs. *Different from young. Age ranged from 18 to 30 y for young and 60 to 83 y for older men. ALM, appendicular lean mass; BM, body mass; HbA1c, glycated hemoglobin; LBM, lean body mass.

### Infusion trials

Most studies were conducted during the daytime, starting in the morning after an overnight fast. A few studies were conducted during the overnight period and started with a standardized dinner 7 h (36, 43) or 5 h (37, 41, 43, 44) prior to the ingestion of a single bolus of intrinsically labeled protein. Three of those overnight studies provided an additional postexercise recovery drink (containing 20 g protein) 2.5 h prior to the ingestion of intrinsically labeled protein (43–44). On the day of the trial, participants came to the laboratory by car or public transport and had a cannula inserted into an antecubital vein for stable isotope–labeled amino acid infusion. For the daytime studies, a second cannula was inserted into a dorsal hand vein of the contralateral arm and placed in a hot-box (60°C) for arterialized blood sampling (51). For the overnight studies, the second cannula was inserted into an antecubital vein of the contralateral arm wrapped in a heated blanket. After collecting a baseline blood sample, the plasma phenylalanine pool was primed with a single dose of l-[ring-^2^H_5_]-phenylalanine (2–3.6 μmol/kg), followed by a continuous intravenous l-[ring-^2^H_5_]-phenylalanine (0.045–0.060 μmol · kg^−1^ · min^−1^) infusion. After ≥2 h of infusion, another blood sample was drawn and participants received a beverage containing intrinsically l-[1-^13^C]-phenylalanine–labeled protein (*t* = 0 min). Blood samples were collected at various time points after protein ingestion. Blood samples were collected in EDTA-containing tubes and centrifuged at 1000 × *g*for 10 min at 4°C. Aliquots of plasma were frozen in liquid nitrogen and stored at −80°C.

### Intrinsically labeled protein

Intrinsically l-[1-^13^C]-phenylalanine–labeled bovine milk protein was produced by infusing l-[1-^13^C]-phenylalanine (420 μmol/min) into lactating Holstein dairy cows as described previously (52–54). Milk was obtained at each milking during and for 24 h following the infusion and stored at −40°C until further processing. Milk was fractionated into a casein and a whey protein concentrate. First, milk was heated to 50°C and skimmed using a cream separator. The skimmed milk was microfiltrated using a membrane with a pore size of 1.4 μm at 50°C to remove microbes. The permeate was filtrated over a 0.2-μm membrane to separate casein micelles (retentate) from soluble whey proteins (permeate) at 55°C. The casein micelles were concentrated, diafiltrated with reverse osmosis (RO) water, and further concentrated to ∼14% dry matter (containing 90% protein). The concentrated casein solution was pasteurized and stored at −20°C until further use. The whey proteins were cooled and concentrated by means of a 10-kDa membrane. The retentate was diafiltrated with RO water and concentrated to an end concentration of ∼5.5% dry matter (containing 96% protein). The whey protein concentrate was sterile filtrated and freeze-dried. For the production of milk protein concentrate, milk was heated to 55°C and skimmed using a centrifuge. The skimmed milk was pasteurized at 75°C for 20 s. The skimmed milk was ultrafiltrated and diafiltrated with RO water using a membrane with a pore size of 20 kDa at 10°C. The permeate was concentrated to ∼18% dry matter (containing ∼80% protein) and spray dried. All protein batches met the chemical and microbiological specifications for human consumption. The amount of protein powder consumed was adjusted for its protein content such that the desired amount of protein was consumed by the participants. The mean enrichment of the intrinsically labeled protein was 33.3 ± 9.6 mole percent excess (MPE).

### Calculations

The ingestion of intrinsically l-[1-^13^C]-phenylalanine–labeled protein, intravenous infusion of l-[ring-^2^H_5_]-phenylalanine, and frequent blood sampling were applied to assess dietary protein digestion and amino acid absorption kinetics. Total and exogenous phenylalanine rate of appearance (R_a_) and plasma availability of dietary protein–derived phenylalanine (i.e., the fraction of dietary protein–derived phenylalanine that appeared in the circulation) were calculated using modified Steele's equations (28, 55):

(1) \begin{eqnarray*} {\rm{Total\ }}{{\rm{R}}_{\rm{a}}} = \frac{{{{\rm{F}}_{{\rm{iv}}}}{\rm{\ }} - {\rm{\ }}\left[ {{\rm{pV\ }} \times {\rm{\ C}}\left( {\rm{t}} \right){\rm{\ }} \times {\rm{\ }}\frac{{{\rm{d}}{{\rm{E}}_{{\rm{iv}}}}}}{{{\rm{dt}}}}} \right]}}{{{{\rm{E}}_{{\rm{iv}}}}\left( {\rm{t}} \right)}}\ \end{eqnarray*}

(2) \begin{eqnarray*} {\rm{Exogenous\ }}{{\rm{R}}_{\rm{a}}} = \frac{{{\rm{Total\ }}{{\rm{R}}_{\rm{a}}}{\rm{\ }} \times {\rm{\ }}{{\rm{E}}_{{\rm{po}}}}\left( {\rm{t}} \right){\rm{\ }} + {\rm{\ }}\left[ {{\rm{pV\ }} \times {\rm{\ C}}\left( {\rm{t}} \right) \times {\rm{\ }}\frac{{{\rm{d}}{{\rm{E}}_{{\rm{po}}}}}}{{{\rm{dt}}}}} \right]}}{{{{\rm{E}}_{{\rm{protein}}}}}}\\ \end{eqnarray*}

(3) \begin{eqnarray*} {\rm{Plasma\ availability\ }} = \left( {\frac{{{\rm{AU}}{{\rm{C}}_{{\rm{Exogenous\ Ra}}}}}}{{{\rm{Ph}}{{\rm{e}}_{{\rm{protein}}}}}}} \right){\rm{\ }} \times {\rm{\ BM\ }} \times {\rm{\ }}100 \end{eqnarray*}

where F_iv_ is the intravenous l-[ring-^2^H_5_]-phenylalanine tracer infusion rate (μmol · kg^−1^ · min^−1^), pV (0.125 L/kg) is the distribution volume, C(t) is the mean plasma phenylalanine concentration between 2 consecutive time points, dE_iv_/dt is the time-dependent change in plasma l-[ring-^2^H_5_]-phenylalanine enrichment, and E_iv_(t) is the mean plasma l-[ring-^2^H_5_]-phenylalanine enrichment between 2 consecutive time points. Exogenous R_a_ represents the rate at which dietary protein–derived phenylalanine enters the circulation, which is a proxy for dietary protein digestion and amino acid absorption. E_po_(t) is the mean plasma l-[1-^13^C]-phenylalanine enrichment between 2 consecutive time points, dE_po_/dt is the time-dependent change in plasma l-[1-^13^C]-phenylalanine enrichment, and E_protein_ is the l-[1-^13^C]-phenylalanine enrichment of the dietary protein. AUC_Exogenous Ra_ represents the AUC of exogenous R_a_, Phe_protein_ is the amount of dietary phenylalanine ingested (μmol), and BM is the participant's body mass. For each batch of protein the phenylalanine content was determined using GC-MS. The average phenylalanine content was 228 μmol/g for whey protein, 317 μmol/g for casein, and 259 μmol/g for milk protein. Plasma phenylalanine availability was calculated for every hour after protein ingestion and presented as cumulative over time. Plasma phenylalanine availability in percentages was multiplied by the dose of protein ingested to obtain the estimated plasma amino acid availability in grams. This calculation assumes that dietary protein–derived phenylalanine is representative for all essential and nonessential amino acids.

### Statistical analysis

Data are expressed as means ± SDs (tables and text) or means ± SEMs (graphs). Differences in participants’ characteristics, peak and time-to-peak exogenous appearance rates, and plasma appearance over the 0–5-h postprandial period between protein types (whey, casein, milk) or between protein doses (T1, T2, T3) were assessed using 1-factor ANOVA followed by post hoc analysis with Bonferroni correction to locate the differences. Differences in participants’ characteristics, peak and time-to-peak exogenous appearance rates, and plasma appearance over the 0–5-h postprandial period between young and older individuals were assessed using independent 2-sample *t* tests. For all comparisons, differences in dietary protein digestion and absorption kinetics and cumulative plasma amino acid appearance were assessed using 2-factor repeated-measures ANOVA followed by post hoc analysis with Bonferroni correction. Statistical significance was set at *P* < 0.05. All calculations were performed using IBM SPSS Statistics version 25.

## Results

The complete dataset of 602 participants showed that 50% ± 14% of dietary protein–derived phenylalanine appeared in the circulation during the 5-h postprandial period. We subsequently assessed whether the type of protein, protein dose, or age affected dietary protein digestion and absorption kinetics and, as such, impacted the amount of dietary protein–derived phenylalanine released into the circulation.

### Type of protein

**FIGURE 1:**
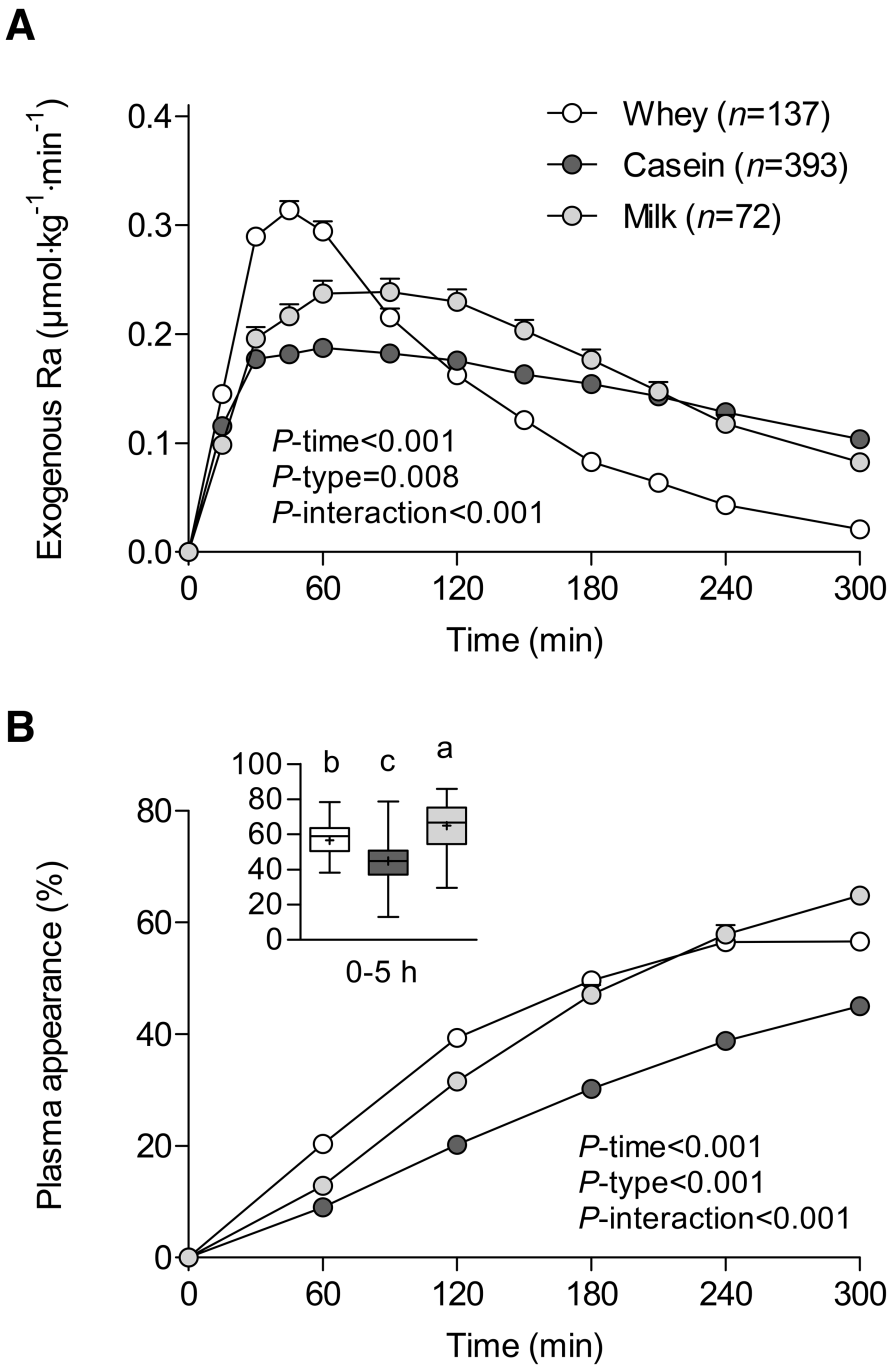
Rate of appearance of dietary protein–derived phenylalanine (A) as well as cumulative plasma phenylalanine appearance over time in percentages (B) and over the entire 0–5-h postprandial period (B, insert) in participants who consumed 23 ± 5, 24 ± 7, and 26 ± 11 g intrinsically labeled whey (*n* = 137), casein (*n* = 393), and milk (*n* = 72) protein, respectively. Values are means ± SEMs. Labeled means without a common letter differ, *P* < 0.05. Ra, rate of appearance.

Appearance rates of dietary protein–derived phenylalanine in the circulation rapidly increased following protein ingestion (*P*-time < 0.001), with marked differences in phenylalanine kinetics between whey, casein, and milk protein ingestion (*P*-interaction < 0.001; **Figure 1**A). Exogenous phenylalanine appearance rates increased to a peak of 0.35 ± 0.09 μmol · kg^−1^ · min^−1^ at 49 ± 20 min after whey protein ingestion compared with 0.25 ± 0.10 μmol · kg^−1^ · min^−1^ at 96 ± 72 min after casein ingestion (*P* < 0.001 for both peak and time-to-peak; Table 1). The ingestion of milk protein resulted in intermediate peak exogenous phenylalanine appearance rates (0.29 ± 0.10 μmol · kg^−1^ · min^−1^), with the time-to-peak being similar to casein ingestion (101 ± 55 min).

The cumulative increase over time in the fraction of dietary protein–derived phenylalanine that appeared in the circulation was higher after the ingestion of whey and milk protein when compared with casein ingestion (*P*-interaction < 0.001; Figure 1B). Moreover, milk protein ingestion resulted in lower plasma phenylalanine appearance during the first 2 h, similar plasma phenylalanine appearance during 3–4 h, and higher plasma phenylalanine appearance after 5 h when compared with whey protein ingestion. Over the entire 5-h postprandial period, the ingestion of casein, whey, and milk protein resulted in a lower (45% ± 11%), intermediate (57% ± 10%), and greater (65% ± 13%) fraction of dietary protein–derived phenylalanine appearing in the circulation, respectively (*P* < 0.001; Figure 1B, insert).

### Whey protein dose

**FIGURE 2:**
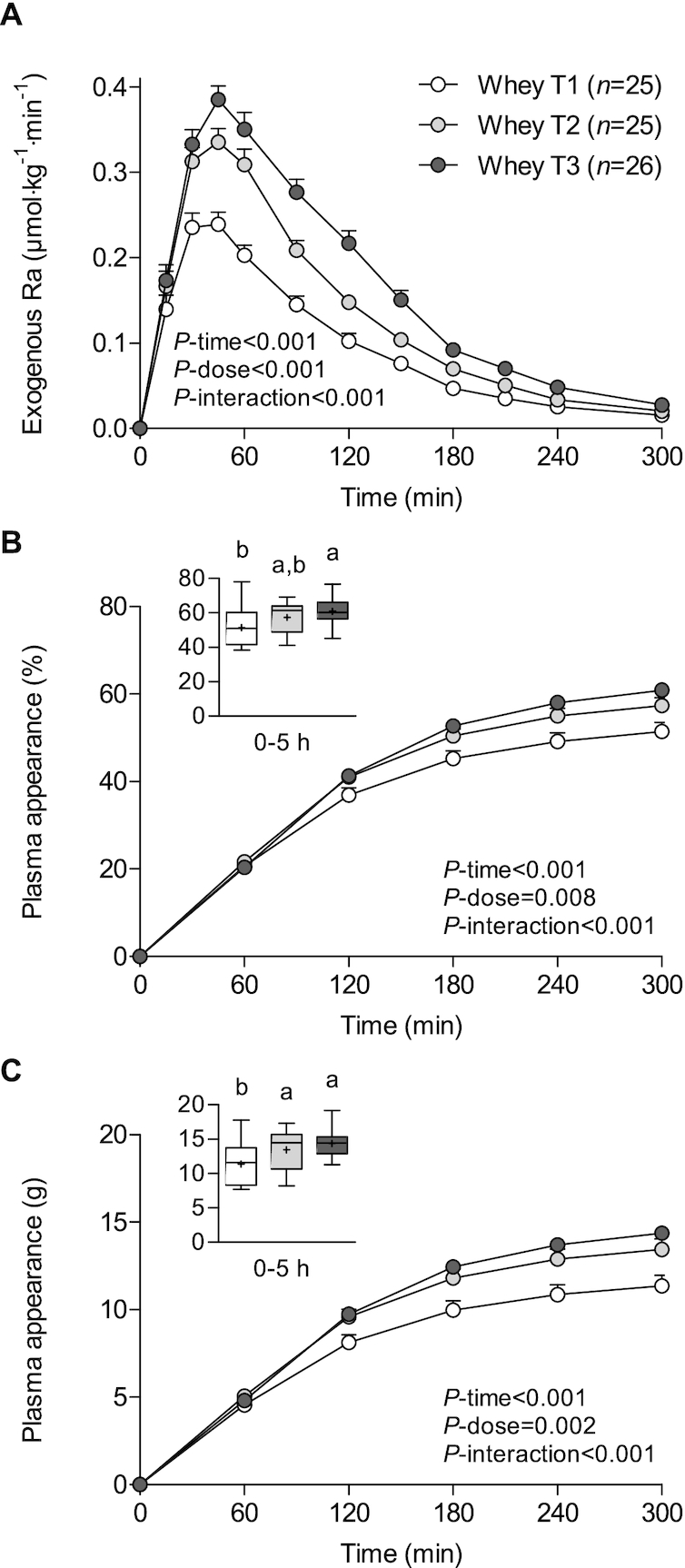
Rate of appearance of dietary protein–derived phenylalanine (A) and cumulative plasma phenylalanine appearance over time in percentages (B), and estimated amount of dietary protein–derived amino acids that appeared in the circulation in grams (C) in participants who consumed intrinsically labeled whey protein (*n* = 76) by tertile of protein dose in grams per kilogram of BM (0.23 ± 0.04 vs. 0.29 ± 0.01 vs. 0.35 ± 0.04 g/kg BM). Values are means ± SEMs. Labeled means without a common letter differ, *P* < 0.05. BM, body mass; Ra, rate of appearance; T, tertile.

We observed a dose–response relation after graded intakes (relative to BM) of whey protein (*P*-interaction < 0.001; **Figure 2**A), with exogenous phenylalanine appearance rates reaching peak values of 0.28 ± 0.07, 0.38 ± 0.06, and 0.43 ± 0.08 μmol · kg^−1^ · min^−1^ after consuming the lower, medium, and higher dose of whey protein (*P* < 0.001; Table 2). Peak exogenous phenylalanine appearance rates were reached at 45 ± 22 min, 43 ± 11 min, and 50 ± 20 min after ingesting the lower, medium, and highest whey protein dose, respectively, with no significant differences between doses (*P* = 0.382).

The cumulative increase over time in the fraction of dietary protein–derived phenylalanine that appeared in the circulation differed between the whey protein doses (*P*-interaction < 0.001), with a greater fraction appearing after ingesting the medium and larger whey protein doses when compared with the lower whey protein dose from 3 h onwards (Figure 2B). Over the 5-h postprandial period, 51% ± 10%, 57% ± 9%, and 61% ± 7% of the dietary protein–derived phenylalanine had appeared in the circulation following the ingestion of the smaller, intermediate, and greater dose of whey protein, respectively (*P* = 0.001; Figure 2B, insert). When calculating the estimated plasma amino acid availability in grams we observed that a greater amount of dietary protein–derived amino acids had appeared in the circulation after ingesting the intermediate and greater doses of whey protein when compared with the smaller dose from 2 h onwards (*P*-interaction < 0.001; Figure 2C). Over the 5-h postprandial period, 11.4 ± 3.0 g, 13.4 ± 3.0 g, and 14.4 ± 1.8 g of the dietary protein–derived amino acids had appeared in the circulation after ingesting the smaller, intermediate, and greater dose of whey protein, respectively (*P* < 0.001; Figure 2C, insert).

### Casein dose

**FIGURE 3:**
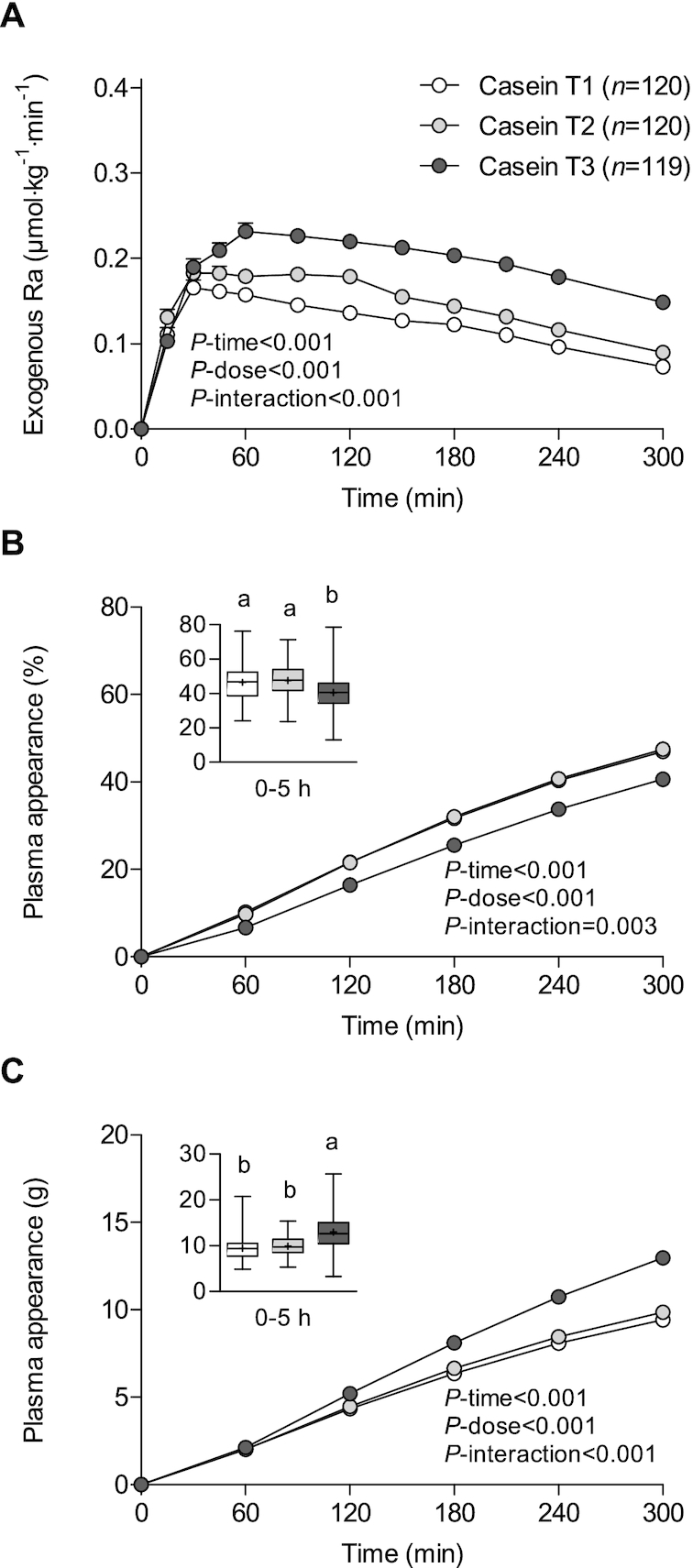
Rate of appearance of dietary protein–derived phenylalanine (A) and cumulative plasma phenylalanine appearance over time in percentages (B) and estimated amount of dietary protein–derived amino acids that appeared in the circulation in grams (C) in participants who consumed intrinsically labeled casein (*n* = 359) by tertile of protein dose in grams per kilogram of BM (0.24 ± 0.01 vs. 0.28 ± 0.02 vs. 0.44 ± 0.07 g/kg BM). Values are means ± SEMs. Labeled means without a common letter differ, *P* < 0.05. BM, body mass; Ra, rate of appearance; T, tertile.

We subsequently assessed the dose–response relation for casein (**Figure 3**) and observed a stepwise increase in exogenous phenylalanine appearance rates with increasing intakes (relative to BM) of casein (*P*-interaction < 0.001; Figure 3A). Peak exogenous phenylalanine appearance rates averaged 0.23 ± 0.09, 0.26 ± 0.10, and 0.29 ± 0.10 μmol · kg^−1^ · min^−1^ for the smaller, intermediate, and greater dose of casein, respectively (*P* < 0.001; Table 3). Time-to-peak was similar between the smaller (91 ± 73 min) and intermediate (87 ± 66 min) dose, but it took longer to reach peak exogenous phenylalanine appearance rates after ingesting the higher dose of casein (116 ± 77 min; *P* = 0.004).

The cumulative increase over time in the fraction of dietary protein–derived phenylalanine that entered the circulation differed between the ingestion of different amounts of casein (*P*-interaction = 0.003; Figure 3B). Casein-derived phenylalanine availability did not differ between the smaller and intermediate dose, but was lower after ingesting the larger dose of casein at all time points. Over the 5-h postprandial period, 47% ± 12% and 47% ± 10% of dietary protein–derived phenylalanine appeared in the circulation after the ingestion of the smaller and intermediate protein doses, with only 41% ± 11% of the larger casein dose entering the circulation (*P* < 0.001; Figure 3B, insert). Despite the lower relative fraction, an absolute greater estimated amount of dietary protein–derived amino acids had appeared in the circulation after ingesting the greater dose when compared with the small and intermediate doses of casein (*P*-interaction < 0.001; Figure 3C). Over the entire 5-h postprandial period, 9.4 ± 2.4 g, 9.9 ± 2.1 g, and 13.0 ± 3.6 g amino acids were calculated to have appeared in the circulation 5 h after ingesting the smaller, intermediate, and greater dose of casein, respectively (*P* < 0.001; Figure 3C, insert).

### Age

**FIGURE 4:**
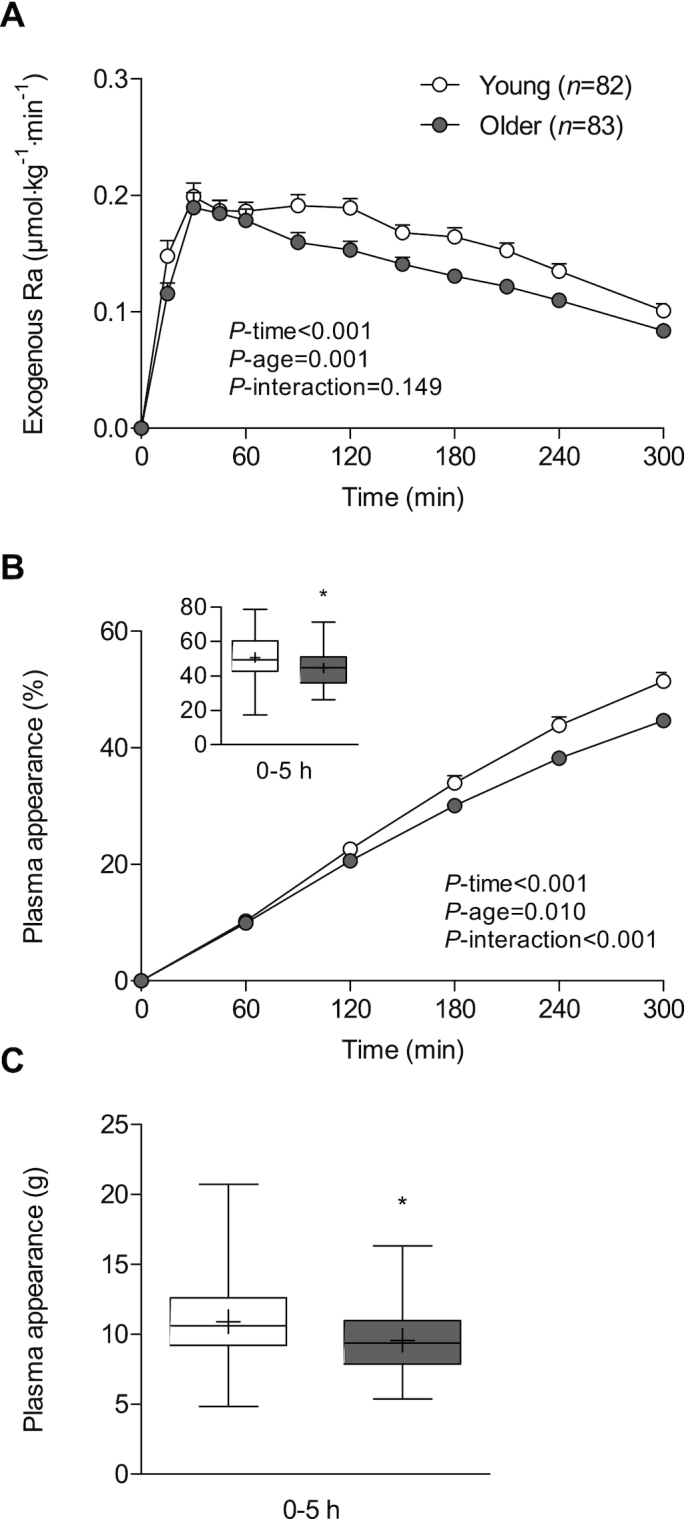
Rate of appearance of dietary protein–derived phenylalanine (A) and cumulative plasma phenylalanine appearance over time in percentages (B) and estimated amount of dietary protein–derived amino acids that appeared in the circulation in grams (C) after the ingestion of 22 ± 5 g intrinsically labeled casein in young (*n* = 82) and older (*n* = 83) men. Values are means ± SEMs. *Different from young men, *P* < 0.05. Ra, rate of appearance; T, tertile.

Last, we investigated whether age affects dietary protein digestion and absorption kinetics and the subsequent appearance of dietary protein–derived phenylalanine in the circulation. We included data from 4 studies from our laboratory in which we compared postprandial protein handling in both young and older individuals. These 4 studies used casein as the type of protein consumed. **Figure 4** shows compromised protein digestion and absorption kinetics in older when compared with young individuals (*P*-age = 0.001, *P*-interaction = 0.149). Peak exogenous phenylalanine appearance rates (0.28 ± 0.10 vs. 0.25 ± 0.11 μmol · kg^−1^ · min^−1^) and time-to-peak (96 ± 70 vs. 84 ± 72 min) did not differ between young and older individuals (*P* = 0.151 and *P* = 0.272, respectively; Table 4). However, the fraction of dietary protein–derived phenylalanine appearing in the circulation was significantly lower in the older compared with the young individuals (*P*-interaction < 0.001; Figure 4B). Over the 5-h postprandial period, 45% ± 10% of dietary protein–derived phenylalanine had appeared in the circulation in the older individuals, which was significantly lower compared with the young individuals (51% ± 14%; *P* = 0.001; Figure 4B, insert). This translated to an estimated 9.6 ± 2.2 g compared with 10.9 ± 2.7 g dietary protein–derived amino acids appearing in the circulation during the 5-h postprandial period in the older versus the young individuals, respectively (*P* = 0.001; Figure 4C). Similar results were obtained when excluding the co-interventions exercise, carbohydrate co-ingestion, and (local) insulin administration.

## Discussion

Our analysis combined data from 18 RCTs with >600 participants and showed that ∼50% of dietary protein–derived phenylalanine appeared in the circulation within a 5-h postprandial period without taking into account the ingested protein amount or type. This suggests that ≥50% of dietary protein is effectively digested and absorbed and becomes available for peripheral tissues such as skeletal muscle, while the other 50% of dietary protein is either not (yet) digested and absorbed, or retained and taken up by splanchnic tissues such as the gut and liver. Postprandial plasma phenylalanine availability gradually increased over time and did not yet reach a plateau at 5 h, suggesting that more dietary protein–derived phenylalanine will appear in the circulation beyond the 5-h postprandial period. Indeed, our studies have shown greater postprandial plasma phenylalanine availability when the postprandial period was extended to 6 h (20, 56) or 7.5 h (41, 43, 44). Interestingly, we observed a large variation in the fraction of dietary protein–derived phenylalanine released into the circulation, ranging between 25% and 80% of the ingested dietary protein. As such, we investigated whether this variability can be explained, at least partly, by the type of protein provided, the dose of protein ingested, and/or the age of the participant.

In line with the current literature (3, 20), we showed that whey protein is more rapidly digested and absorbed when compared with micellar casein. Few studies have assessed protein digestion and absorption kinetics after ingesting intact bovine milk protein, which is composed of both whey protein and micellar casein in a ratio of 20:80 (29, 56, 57). Here, we show that ingested milk protein concentrate is more rapidly digested and absorbed when compared with micellar casein, but more slowly digested and absorbed when compared with whey protein. Interestingly, when the entire 5-h postprandial period was taken into account, a greater fraction of dietary protein–derived phenylalanine appeared in the circulation after milk protein ingestion (65% ± 13%) when compared with whey protein (57% ± 10%) or casein (45% ± 11%) ingestion. These findings indicate that ingestion of a dietary protein that is most rapidly digested and absorbed, such as whey protein, does not necessarily result in the greatest plasma availability of dietary protein–derived phenylalanine. In fact, it has previously been suggested that the more rapid digestion of soy protein when compared with milk protein resulted in more amino acids being directed towards deamination pathways and liver protein synthesis (58). However, the differences in amino acid composition and Digestible Indispensable Amino Acid Score between protein types may also impact splanchnic amino acid uptake and subsequent plasma amino acid availability. Overall, it is evident that the type of ingested protein is one of the main factors that modulate postprandial availability of dietary protein–derived phenylalanine in the circulation.

We subsequently evaluated whether the ingestion of various dosages of both whey protein and micellar casein affected dietary protein digestion and phenylalanine absorption kinetics and subsequent increase in dietary protein–derived phenylalanine availability in the circulation. With increasing doses of whey protein, we observed a stepwise increase in protein digestion and phenylalanine absorption rates (Figure 2A). Higher postprandial release rates of dietary protein–derived phenylalanine were accompanied by a greater availability of dietary protein–derived phenylalanine in the circulation. After consuming the higher dose of whey protein, 61% ± 7% of the dietary protein–derived phenylalanine had appeared in the circulation throughout the 5-h postprandial period, compared with 51% ± 10% after ingesting the lower whey protein dose. This translates to an estimated amount of 14.4 ± 1.8 versus 11.4 ± 3.0 g amino acids that appeared in the circulation over 5 h after ingesting the high- versus the low-protein dose, respectively. For the casein dose–response, we also observed a stepwise increase in appearance rates of dietary protein–derived phenylalanine (Figure 3A). However, higher rates of protein digestion and phenylalanine absorption after the higher protein dose did not result in greater postprandial plasma phenylalanine availability (Figure 3B). After consuming the higher doses of casein, merely 41% ± 11% of protein-derived phenylalanine had appeared in the circulation, compared with 47% ± 12% after consuming the lower protein dose. Nonetheless, a greater absolute amount of dietary protein–derived amino acids appeared in the circulation after consuming the higher (13.0 ± 3.6 g) compared with the lower (9.4 ± 2.4 g) casein dose. These findings are in line with recent work from our laboratory assessing the milk protein dose–response (15–30–45 g) during recovery from resistance-type exercise in older men (56). This study showed that lower relative but greater absolute amounts of protein-derived amino acids appeared in the circulation after consuming a higher dose of milk protein throughout a 6-h postprandial period (56). The discrepancy between whey protein and micellar casein in the current study may be attributed to the duration of the postprandial period. Whey protein at the various ingested doses is almost completely digested and absorbed (i.e., exogenous R_a_ at 5 h is close to zero), whereas casein does not seem completely digested and absorbed yet within the 5-h postprandial period. As such, casein-derived phenylalanine may likely continue to appear in the circulation after the 5-h postprandial period. Overall, our data indicate that the availability of dietary protein–derived phenylalanine in the circulation increases with increasing the ingested protein dose and also depends on the duration of the postprandial period that is assessed.

To define the impact of age on dietary protein digestion and phenylalanine absorption kinetics and the subsequent availability of dietary protein–derived phenylalanine in the circulation, we combined data from 4 RCTs that compared young and older individuals within the same study (27, 32, 35, 42). Peak protein digestion and phenylalanine absorption rates were similar between young and older men. However, protein digestion and phenylalanine absorption rates declined more rapidly in older when compared with the young individuals, which resulted in a lower postprandial availability of dietary protein–derived phenylalanine in the circulation. While 51% ± 14% of dietary protein–derived phenylalanine had appeared in the circulation over the 5-h postprandial period in the young men, 45% ± 10% appeared in the circulation in older men. This translates to an estimated amount of 10.9 ± 2.7 g when compared with 9.6 ± 2.2 g of dietary protein–derived amino acids (of the ∼22 g ingested) that appeared in the circulation over the 5-h postprandial period in young compared with older men, respectively. Such a seemingly small impairment in dietary protein digestion and absorption with each meal at an older age could have a substantial impact on muscle mass maintenance in the long term. Data from the current study suggest that ingesting a larger protein dose and selecting milk protein as the preferred protein type may result in greater postprandial plasma phenylalanine availability, which may compensate for the lower postprandial phenylalanine availability at a more advanced age.

A potential limitation of this study is that mainly men were included in the analysis. However, we have recently shown that dietary protein digestion and phenylalanine absorption kinetics and the subsequent postprandial plasma phenylalanine availability do not differ between women and men (59). Another limitation is that we do not have data on habitual protein intake amounts prior to all experiments. We have previously shown that 2 wk of habituation to a lower protein intake (i.e., 0.7 g · kg^−1^ · d^−1^) compared with a higher protein intake (i.e., 1.5 g · kg^−1^ · d^−1^) augments the postprandial availability of dietary protein–derived phenylalanine in the circulation (21). Although we expect a low(er) variation in habitual protein intake amounts between the participants in the included studies, determining habitual protein intake amounts may need to be considered in future studies.

In conclusion, protein type, protein dose, and age modulate dietary protein digestion and phenylalanine absorption kinetics and, as such, the postprandial release of dietary protein–derived phenylalanine into the circulation in vivo in humans. Older age is accompanied by an attenuated postprandial release of dietary protein–derived phenylalanine into the circulation.

## Notes

Supported by Top Institute Food and Nutrition, which is a public–private partnership on precompetitive research in food and nutrition.

Author disclosures: LJCvL and LBV have received research grants, consulting fees, speaking honoraria, or a combination of these, from Friesland Campina, Nutricia Research, and PepsiCo. The other authors report no conflicts of interest.

The industrial partners contributed to the project through regular discussions.

Abbreviations used: BM, body mass; R_a_, rate of appearance; RCT, randomized controlled trial; T, tertile.
